# Case Report: Severe ANCA-positive eosinophilic granulomatosis with polyangiitis presenting with Loeffler endocarditis and cryofibrinogenemia-associated digital gangrene successfully treated with rituximab

**DOI:** 10.3389/fimmu.2026.1882906

**Published:** 2026-07-07

**Authors:** Irena Ostric Pavlovic, Danijela Trifunovic-Zamaklar, Branka Bonaci-Nikolic, Mileva Bascarevic, Vesna Tomic-Spiric, Aleksandra Peric-Popadic, Natasa Dragasevic Miskovic, Boris Ukropina, Aleksandar Reljic, Natasa Kusic, Andrija Pavlovic, Antonije Velickovic, Sara Radovic, Snezana Arandjelovic

**Affiliations:** 1 Clinic of Allergology and Immunology, University Clinical Centre of Serbia, Belgrade, Serbia; 2 School of Medicine, University of Belgrade, Belgrade, Serbia; 3 Clinic of Cardiology, University Clinical Centre of Serbia, Belgrade, Serbia; 4 Clinic of Neurology, University Clinical Centre of Serbia, Belgrade, Serbia; 5 Clinic of Orthopedics, University Clinical Centre of Serbia, Belgrade, Serbia; 6 Department of Cardiology, University Children’s Clinic, Belgrade, Serbia

**Keywords:** ANCA-associated vasculitis, cryofibrinogenemia, digital gangrene, eosinophilia, eosinophilic granulomatosis with polyangiitis, Loeffler endocarditis

## Abstract

**Background:**

Eosinophilic granulomatosis with polyangiitis (EGPA) is an anti-neutrophil cytoplasmic antibody (ANCA)-associated systemic vasculitis characterized by asthma, eosinophilia and inflammation of small-tomedium vessels. Although respiratory and neurological manifestations are predominant, cardiovascular involvement and ischemic complications may significantly affect the prognosis.

**Case presentation:**

We report a case of a 37-year-old woman, diagnosed with EGPA based on a history of bronchial asthma, marked eosinophilia, mononeuritis multiplex progressing to sensorimotor polyneuropathy, nasal polyps and vasculitic lesions of the toes and feet associated with MPO-ANCA positivity. Despite treatment with high dose corticosteroids, cyclophosphamide, anticoagulation, and plasmapheresis, the disease progressed with development of digital necrosis and ischemic lesions in the spleen, kidney, and cerebrum. Transesophageal echocardiographic examination confirmed Loeffler endocarditis of the mitral and aortic valve. Due to the fulminant and therapeutically challenging disease course, treatment with rituximab (375mg/m^2^) was initiated, with positive clinical and biochemical response. After clear demarcation, amputation of the affected toes was successfully performed. Maintenance therapy included corticosteroids, methotrexate (later discontinued due to hepatotoxicity), and antimalarials, with sustained remission during a three-year follow-up.

**Conclusion:**

To our knowledge, this rare form of EGPA presenting as a combination of digital gangrene and Loeffler endocarditis associated with MPO-ANCA positivity and cryofibrinogenemia has not previously been reported. The case highlights the potential immunopathogenic interplay between eosinophilic inflammation, ANCA-mediated vasculitis, and cryofibrinogenemia-associated thrombosis leading to severe ischemic complications. Early recognition and timely initiation of B-cell–targeted therapy with rituximab resulted in sustained remission and functional recovery.

## Introduction

Eosinophilic granulomatosis with polyangiitis (EGPA), formerly known as Churg-Strauss syndrome, is the rarest ANCA-associated vasculitis (AAV), with an estimated incidence of approximately 2 cases per million person-years and prevalence of about 34 cases per million individuals (1, 2). The disease typically manifests in early to middle adulthood, and is characterized by asthma, peripheral eosinophilia and necrotizing inflammation of small-to-medium blood vessels (2, 3).

EGPA pathogenesis is driven by a type-2 immune response mediated by Th2 lymphocytes and type-2 innate lymphoid cells producing IL-4, IL-5 and IL-13 (3, 4). Environmental triggers and allergens amplify this pathway through epithelial alarmins, such as thymic stromal lymphopoietin, promoting eosinophil proliferation, survival and tissue infiltration. IL-5 represents the central effector cytokine regulating eosinophilopoiesis and eosinophil-mediated cytotoxicity, responsible for organ damage, particularly affecting the respiratory tract and cardiovascular system (4, 5).

In a subset of patients, persistent eosinophilic inflammation is associated with activation of adaptive immunity and production of ANCA specific to myeloperoxidase. ANCA-mediated neutrophil activation and complement engagement induce endothelial injury and necrotizing vasculitis (3, 4). Although ANCA antibodies are detected in only 30–40% of patients, they define a distinct immunological phenotype associated with peripheral neuropathy and renal involvement, whereas ANCA-negative disease more commonly presents with eosinophilic tissue infiltration including cardiac manifestations, such as Loeffler endocarditis (3, 4, 6).

The heterogeneity of clinical manifestations supports the concept of EGPA as dynamic disease spectrum in which eosinophilic inflammation and autoantibody-mediated vasculitis coexist rather than represent separate entities (3–5, 7). However, severe ischemic complications (including gangrene and multiorgan infarctions) cannot be fully explained by either mechanism alone, suggesting the involvement of an additional prothrombotic process.

Activated B-cells may contribute to development of cryofibrinogenemia, a condition characterized by cold-induced precipitation of fibrinogen-containing complexes within small vessels, potentially resulting in vascular occlusion and tissue ischemia (8–10). Although cryofibrinogenemia has rarely been reported alongside ANCA-associated vasculitis, immune-mediated endothelial injury may indicate a shared immunopathogenic mechanism rather than a coincidental association. However, it still remains unclear whether cryofibrinogenemia represents a true pathogenic contributor of the vascular injury or an epiphenomenon of the systemic immune activation.

Here, we report a patient with an unusual overlapping phenotype of EGPA, characterized by eosinophilic cardiac involvement, MPO-ANCA-mediated vasculitis, and cryofibrinogenemia, presenting with digital gangrene and Loeffler endocarditis.

## Case report

A 37-year-old female patient was referred to the emergency department due to intense pain in the right foot after an accidental fall in the bathtub. She was examined by an orthopedic surgeon followed by the X-ray of the right ankle that showed no evidence of fracture. The foot was immobilized with fixation bandages, and afterwards physical therapy was initiated.

Due to lingering pain and swelling of the right foot with elevated D-dimer levels the patient was referred to vascular surgeon. Color doppler scan (CDS) of the lower extremities was performed, ruling out vascular thrombosis. Other routine laboratory tests, including a complete blood count were not performed at that time. Over the following two months, she was treated with low-molecular-weight heparin (LMWH). As the symptoms persisted and the pain intensified, magnetic resonance imaging (MRI) of the right ankle was performed, suggesting a possible diagnosis of Sudeck’s disease (complex regional pain syndrome type I).

During the following weeks, she had progressively worsening paresthesia, muscle weakness, cold-induced symptoms (such as liveoid maculas on both feet),and pain in the lower extremities, resulting in significant impairment of functional status. Despite being previously physically active, she gradually required assistance for ambulation, progressed to wheelchair use, and ultimately became bedridden.

**Figure 1:**
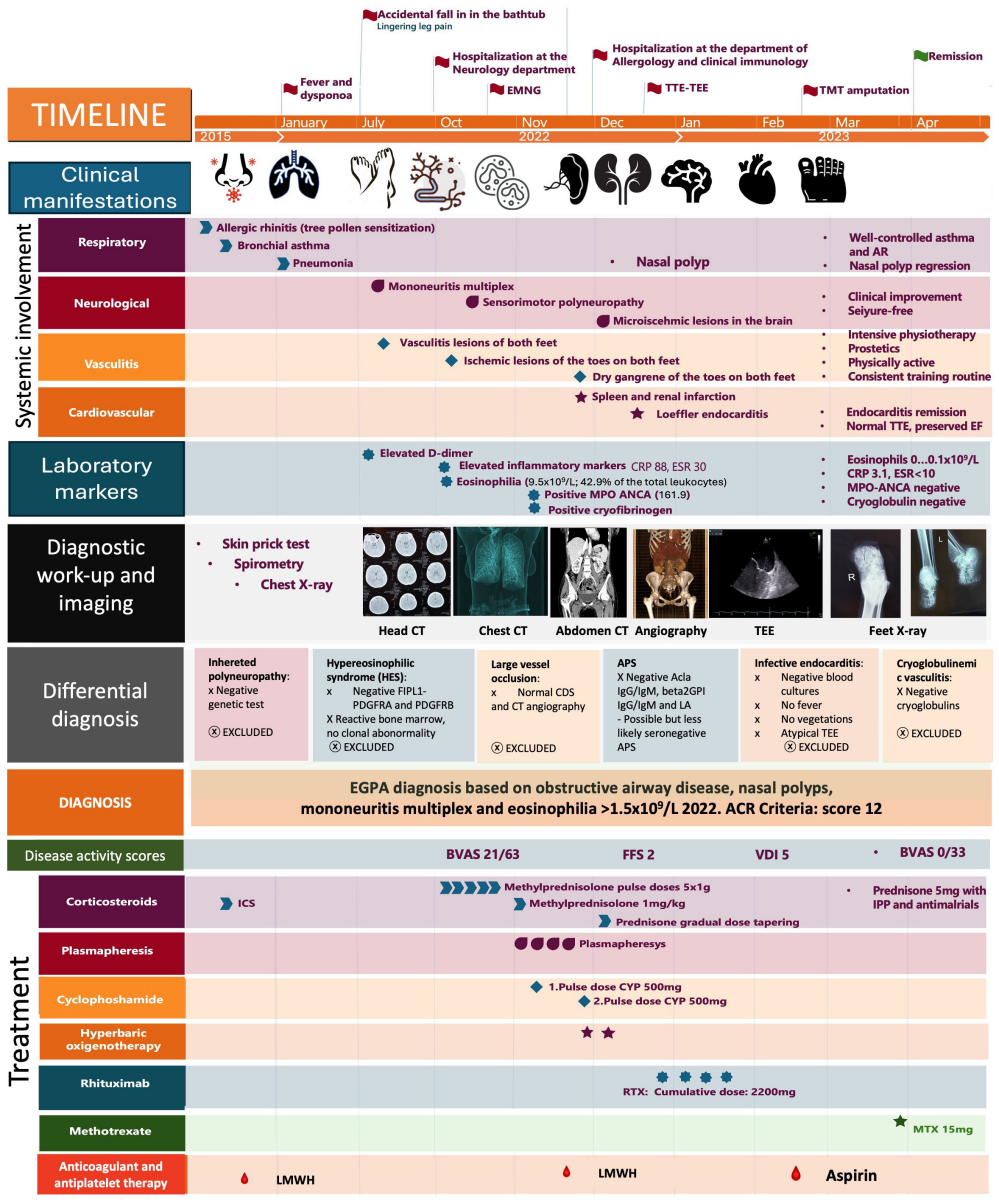
Chronological evolution of the clinical manifestations, laboratory findings, diagnostic evaluation, differential diagnosis, disease activity scores, treatment and outcome in a patient with severe form of EGPA.

Consequently, she was admitted to the emergency neurology department (timeline showed in **Figure 1**).

Computed tomography (CT) of the brain and cervical spine were normal, ruling out an acute neurological event. Electromyoneurography (EMNG) demonstrated a sensorimotor primary axonal polyneuropathy, with marked asymmetry between the upper extremities and symmetrical findings in the lower extremities, which correlated with the neurological examination, and led to the hospitalization at the department of Neurology at the University Clinical Centre of Serbia.

Her past medical history revealed allergic rhinitis (with known sensitization to grass and tree pollen) and mild bronchial asthma (since 2015),which has been well controlled with low-dose inhaled corticosteroids (ICS), bronchodilators. There was no relevant family history. The patient was a nonsmoker and denied the use of psychoactive substances.

Upon the physical examination, the patient was conscious and fully oriented, eupnoic at rest, acyanotic, and anicteric. Pulmonary auscultation revealed normal vesicular breath sounds bilaterally, without other pathological findings. Cardiovascular examination demonstrated a regular heart rhythm with normal heart sounds and no audible murmurs. Blood pressure was 120/75 mmHg. The abdomen was scaphoid, soft, and non-tender on palpation. Examination of the lower extremities revealed oedema of the right foor and livedoid vasculopathy on both feet. There were no other active cutaneous lesions on the rest of the skin, and no signs of active hemorrhagic syndrome.

Laboratory evaluation demonstrated marked peripheral blood eosinophilia (9.5 ×10^9^/L; 42% of total leukocytes). Therefore, detailed hematological evaluation was performed to exclude primary or clonal causes of hypereosinophilia. Hypereosinophilic syndrome (HES) and eosinophilic hematologic malignancies were considered in the differential diagnosis. Peripheral blood lymphocyte immunophenotyping revealed an increased eosinophil concentration (30%) without atypical T-lymphocyte populations. Bone marrow examination revealed reactive medullary hypereosinophilia without morphological abnormalities. Molecular cytogenetic analysis, using fluorescence *in situ* hybridization (FISH) showed no evidence of rearrangements associated with clonal eosinophilic disorders (FISH analysis was negative for the CHIC2 deletion/FIP1L1-PDGFRA fusion and for rearrangements of the PDGFRB gene).

Among other laboratory findings, C-reactive protein (CRP) was elevated (88.3 mg/L- reference range 0-5mg/L 5mg/L) and erythrocyte sedimentation rate (ESR) was high 30mm/h (reference range<10mm/h). Other results of the routine blood chemistry test were within normal limits (key laboratory findings are displayed in **Table 1**).

**Table 1:** Key laboratory, immunologic, coagulation, and cardiac parameters at diagnosis and after rituximab therapy.

Parameter	At diagnosis	After rituximab	Reference range
Leukocyte count	**22.7 ×10^9^/L**	**13.9 ×10^9^/L**	4–10 ×10^9^/L
Eosinophil count	**9.5 ×10^9^/L**	0.0×10^9^/L	<0.5 ×10^9^/L
Eosinophils (%)	**42%**	0%	<5%
CRP	**88.3 mg/L**	2.1mg/L	<5 mg/L
ESR	**30 mm/h**	4 mm/h	<10 mm/h
Hemoglobin	**115 g/L**	138 g/L	120–160 g/L
Platelet count	**536×10^9^/L**	347 ×10^9^/L	150–400 ×10^9^/L
D-dimer	**3.59mg/L**	0.23 mg/L	<0.5 mg/L
fibrinogen	4.1g/dl	3.0 g/dl	1.7/4.2g/dl
aPTT	21	22.3	21.6-28.7s
MPO-ANCA	**161 U/mL**	Negative	<5 U/mL
PR3-ANCA	Negative	Negative	<5 U/mL
Cryofibrinogen	**Positive (+)**	Negative	Negative
Cryoglobulins	Negative	Negative	Negative
Cold agglutinins	Negative	Negative	Negative
aCL IgG	<2	<2	0-12 U/ml
aCL IgM	<2	<2	0-12 U/ml
Anti-β2GPI IgG	<2	<2	0-20 U/ml
Anti-β2GPI IgM	<2	<2	0-50 U/ml
Lupus anticoagulant	Negative	Negative	Negative
Troponin	**59 ng/L**	**35**	<14 ng/L
NT-proBNP	**5688 pg/mL**	**340**	<125 pg/mL

Values outside the reference range are marked in bold.

Immunological testing revealed normal serum immunoglobulin levels or complement components. However, extended immunological screening demonstrated positive antineutrophil cytoplasmic antibodies (ANCA) with markedly elevated anti-myeloperoxidase antibodies (MPO-ANCA 161 U/mL; reference range <5 U/mL), whereas anti-proteinase 3 antibodies (PR3-ANCA) were within the normal range. The semiquantitative cryofibrinogen test was positive (+) (performed at the Blood Transfusion Institute of Serbia) while cryoglobulins and cold agglutinins were not detected. Laboratory results are presented on Microbiological analyses were negative.

Complete diagnostic algorithm performed in this case is represented on **Table 2**.

**Table 2:** Multidisciplinary diagnostic approach in a patient with severe EGPA.

Baseline laboratory investigations	Immunological and hematological tests	Imaging studies	Other procedures
Complete blood count	ANCA IIF and ELISA	Chest radiograph	Pulmonary function tests Spirometry Gas diffusion test
Routine serum chemistries	IgG, IgA, IgM, IgE	HRCT body with angiography	Dermatological evaluation
Inflammatory markers CRP, ESR, fibrinogen	Cryofibrinogen	Head CT	EMNG
Hemostasis PT, aPTT, D-dimer	Cryoglobulins	Paranasal sinus CT	ENT evaluation
Cardiac markers BNP, troponin, CK	Cold agluttinins	Color Doppler of the upper and lower limbs	Vascular surgeon evaluation
Urinalysis, 24-h proteinuria, UPCR	Hematological evaluation	Echocardiography TTE and TEE	Cardiology evaluation ECG, BP and HR monitoring
Mycrobiological tests: Fecal parasite test Serological analysis for Toxoplasma, Toxocara, Echinococcus HBV, HCV and HIV tests	Blood smeer Immunofenotipisation of the peripherial lymphocites FISH	Cardiac magnetic resonance	

Based on the 2022 American College of Rheumatology/European Alliance of Associations for Rheumatology (ACR/EULAR) classification criteria, a diagnosis of eosinophilic granulomatosis with polyangiitis (EGPA) was established (score 12) (6). The diagnosis was supported by the presence of vasculitic skin lesions, obstructive airway disease, nasal polyposis, mononeuritis multiplex, and peripheral eosinophilia (>1.5 ×10^9^/L). At the time of initial evaluation, disease activity assessed using the Birmingham Vasculitis Activity Score (BVAS) was 21/63.

The patient was initially treated with pulse therapy consisting of intravenous methylprednisolone (1 g daily for 5 days), followed by oral prednisone at a dose of 1 mg/kg/day). In addition, four sessions of plasmapheresis were performed. These interventions did not result in significant clinical improvement, except for a transient reduction in peripheral eosinophil counts and inflammatory markers, so two cyclophosphamide pulses (cumulative dose 1000 mg) were administered.

Meanwhile, peripheral vascular changes continued to progress, culminating in the development of dry gangrene of the toes. Repeated Doppler ultrasonography and CT angiography demonstrated preserved flow in major arterial vessels, without evidence of significants stenosis or occlusion. The discrepancy between progressive distal ischemic injury and preserved large-vessel patency and favored a presence of mycoangiopathy, with predominant microvascular thrombo-inflammatory phenotype. Besides the initially elevated D-dimer level, hemostasis (PT, INR) was normal. Other potentially contributing thrombophilic conditions were also considered. Repeated immunological testing was performed revealing negative antiphospholipid antibodies (anticardiolipin antibodies (aCL) IgM and IgG, anti-beta-2 glycoprotein 1 antibodies (anti-β_2_GPI) IgM and IgG, and lupus anticoagulant (LA)), but do not rule out seronegative APS. There was no evidence of inherited thrombophilia upon screening for MTHFR; factor V Leiden, factor II mutations, protein C and S. The positive cryofibrinogen test raised the possibility of cryofibrinogenemia contribution to platelet activation induced by epithelial injury leading the development of gangrenous lesions in this severe presentation of ANCA-associated vasculitis (AAV).

Due to ischemic lesions, hyperbaric oxygen therapy (HBO) was initiated. Following the second HBO session, the patient developed an episode of impaired consciousness and bilateral tonic-clonic convulsion, representing a new neurological event. Brain computed tomography (CT) revealed acute *de novo* microischemic lesions, which were subsequently confirmed by magnetic resonance imaging (MRI). Antiepileptic therapy was initiated, after which no further seizures occurred.

Given the presence of peripheral and cerebral microangiopathic lesions, together with newly detected ischemic lesions in the spleen and kidneys confirmed by abdominal CT, despite continuous anticoagulation therapy (confirmed by anti Xa within the reference range: 0.80-0.96), further evaluation for a potential thromboembolic source was undertaken.

**Figure 2:**
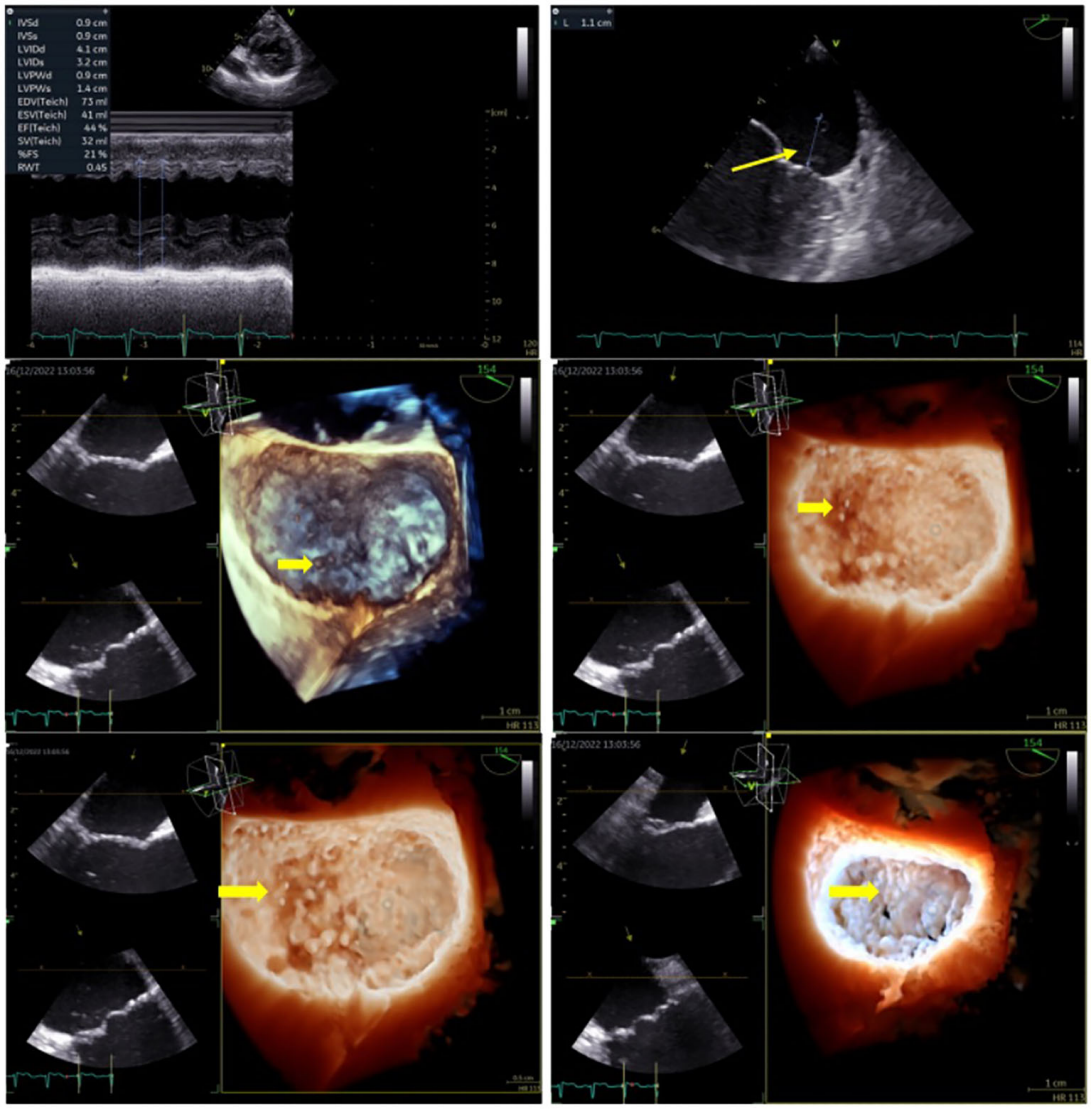
Transesophageal echocardiography findings consistent with Loeffler endocarditis.

The initial consideration was development of the infective endocarditis (IE). The embolization pattern of the central nervous system emboli, spleen and kidney ischemia (revealed on body CT) and peripheral embolization of the feet and toes was consistent with bacterial endocarditis of the left heart. However, based on the series of negative blood cultures, lack of fever and microbiological confirmation IE was subsequently ruled out. Both transthoracic (TTE) and transesophageal echocardiography (TEE) were performed, but the findings were inconsistent with infectious vegetations. TEE revealed filamentous platelet-rich aggregates on the mitral and, to a lesser extent, on the aortic valve, with marked morphological alterations (distinct “hairy” appearance of the endocardium). (**Figure 2**). Based on the echocardiographic findings and clinical context, the cardiologist concluded that these changes were most consistent with Loeffler endocarditis associated with EGPA. Although the estimated thromboembolic risk was considered relatively low, anticoagulation therapy with therapeutic doses of low-molecular-weight heparin (LMWH) was continued.

“Pearl-like” filamentous thrombotic aggregates attached to the mitral and aortic valves, and “hairy” endocardium (arrows) compatible with Loeffler endocarditis associated with EGPA.

The patient later became tachycardic and hypertensive, accompanied by elevated cardiac biomarkers (troponin 59 ng/L and NT-proBNP 5688 pg/mL). and a reduced left ventricular ejection fraction (44%). Therefore, cardiovascular magnetic resonance imaging (CMR) was performed. CMR showed no evidence of additional myocardial, pericardial, or large-vessel involvement.

However, the documented cardiovascular manifestation in the form of Loeffler endocarditis increased the Five-Factor Score (FFS) of ANCA-associated vasculitis from 0 to 2, indicating a poor prognosis with an estimated 5-year survival rate of 54.1% (relative risk 2.40).

Follow-up CT scans of the chest and abdomen demonstrated no infiltrative pulmonary changes or granulomatous involvement, while CT angiography was unchanged. However, clear signs of peripheral ischemia with progressive dry gangrene persisted. The presence of necrotic tissue likely contributed to sustained immune activation and ongoing vascular inflammation.

Despite high-dose glucocorticoids, plasmapheresis, cyclophosphamide pulses and anticoagulation, and two the patient experienced ongoing progression of ischemic and neurological manifestations. Given the rapidly evolving organ-threatening disease, escalation to biological treatment was considered necessary before completion of a standard cyclophosphamide induction regimen.

Based on current guidelines and available literature, anti-CD20 therapy was selected as the most appropriate treatment strategy for this phenotype of severe ANCA-positive EGPA with predominant cardiovascular and neurological involvement, in the absence of severe asthma. Rituximab was administered according to the standard dosing regimen (375 mg/m² weekly for four weeks), with a cumulative dose of 2.2 g. The therapeutic response was excellent, with no further signs of disease progression or exacerbation. Following treatment, anti-MPO antibodies and cryofibrinogen became undetectable, and the eosinophil count decreased to 0.1 × 10^9^/L.

**Figure 3:**
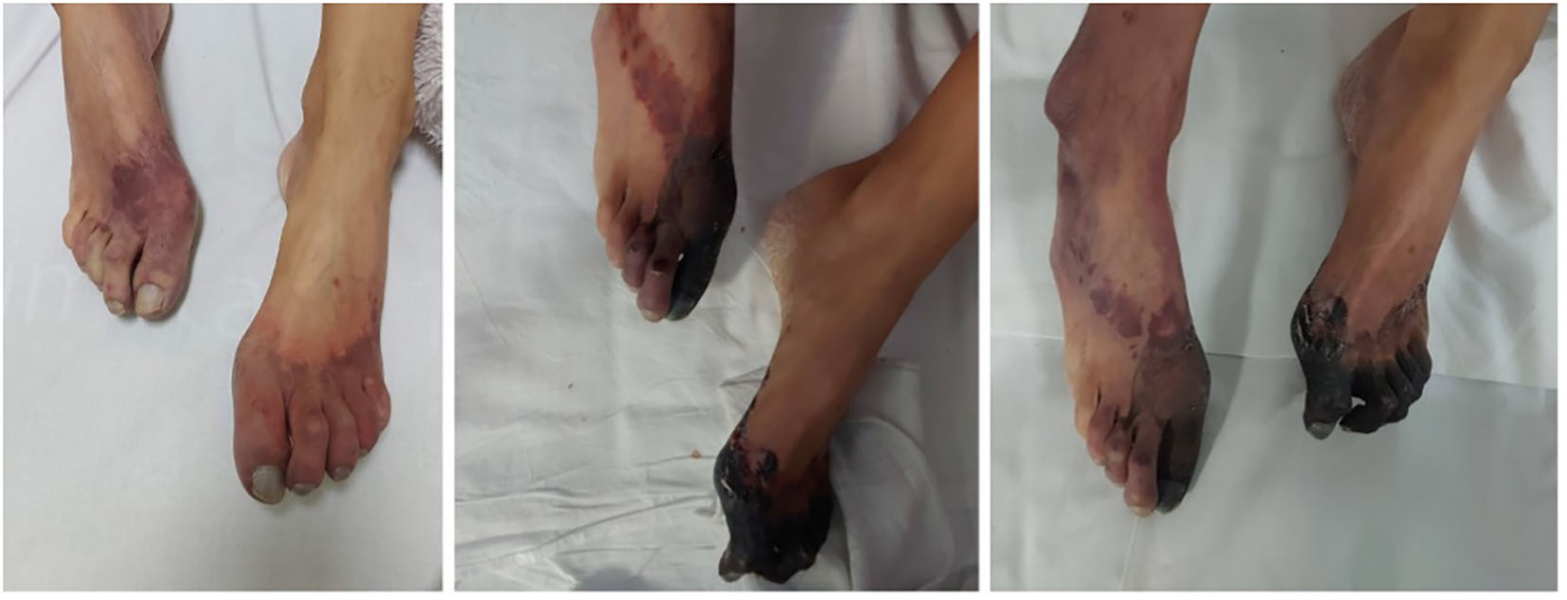
Progressive ischemic necrosis of the feet associated with vasculitis and cryofibrinogenemia.

Secondary hypogammaglobulinemia (IgG level: 3.5 g/L) was later detected, most likely as a consequence of the combined immunosuppressive therapy. A single dose of 20 g of intravenous immunoglobulin was administered. The only persistent complication was progressive gangrene of the feet (**Figure 3**). After a clear demarcation line developed, the patient underwent bilateral transmetatarsal amputation at the orthopedic clinic. At that time, the Vascular Damage Index (VDI) was 5. The postoperative course was uneventful, and the surgical 202 wounds healed without complications.

Following hospital discharge, oral prednisone was gradually tapered to 5 mg once daily. Methotrexate(MTX) was introduced as maintenance therapy at a dose of 15 mg weekly and initially provided stable disease control. However, MTX was discontinued shortly after due to hepatotoxicity. The patient was subsequently maintained on low-dose corticosteroids in combination with antimalarial therapy (hydroxychloroquine 200 mg daily) and antithrombotic therapy (aspirin 100 mg daily), vitamin D3 2000U/day and Trimetoprim sulphomethaxole prophylaxis, with no signs of disease reactivation. Repeated control echocardiographic examination was normal, without signs of endocardial involvement and thrombosis, with improved EF. The BVAS was 3/33 at the end of the first year of follow-up and decreased to 0/33 during the second year.

## Patient perspective

The patient underwent extensive physical rehabilitation and was fitted with foot prostheses, which significantly improved her daily functioning and quality of life. During the three-year follow-up period, the patient remained clinically stable and free of disease exacerbations, with normal laboratory and immunoserological results and with excellent symptom control, physically active and highly motivated.

## Discussion

EGPA is a rare form of ANCA-associated vasculitis characterized by marked clinical heterogeneity (1, 2). This variability reflects two overlapping pathogenic mechanisms: eosinophil-mediated tissue injury driven by type 2 inflammation and ANCA-associated vasculitis caused by B-cell–mediated autoimmunity (3–5). The relative predominance of these immunopathologic pathways, influenced by genetic susceptibility and environmental triggers, determines the clinical phenotype, pattern of organ involvement, and response to therapy.

This case illustrates a rare and severe overlap phenotype characterized by the coexistence of three potentially interacting pathogenic mechanisms: eosinophil-mediated tissue injury, ANCA-associated vasculitis, and cryofibrinogenemia-associated thrombosis. Rather than representing independent complications, these mechanisms may have contributed to this multifactorial thrombo-inflammatory process, resulting in extensive vascular injury.

## Pathophysiological background of EGPA

EGPA typically evolves through three clinical phases (1, 2). In the early phase, tissue damage is largely mediated by eosinophil degranulation products such as major basic protein and eosinophil cationic protein (11). These cytotoxic mediators cause endothelial injury and endomyocardial damage. The development of Loeffler endocarditis in this patient represents the most severe manifestation of eosinophilic cardiac involvement and reflects advanced eosinophil-mediated tissue injury. Endocardial damage promotes thrombus formation and may serve as a source of systemic embolization.

Persistent endothelial injury can subsequently promote activation of adaptive immunity. In a subset of patients, B-cell activation leads to the production of myeloperoxidase-ANCA (MPO-ANCA), resulting in neutrophil activation, complement engagement, and necrotizing small-vessel vasculitis. The neurological manifestations observed in this patient, including mononeuritis multiplex, are consistent with ANCA-mediated ischemic injury of the vasa nervorum.

However, the severity of ischemic complications observed in this case, including digital gangrene and multiorgan infarctions, exceeded what is typically expected from vasculitis alone. Cardiac involvement in EGPA, including Loeffler endocarditis, has been described in the literature, and rare cases of EGPA associated with cryoglobulinemia have also been reported (12).

## Role of cryofibrinogenemia in ischemic complications

Cryofibrinogenemia is characterized by cold-induced precipitation of fibrinogen-containing complexes within small vessels, leading to vascular occlusion, purpura, tissue necrosis, and gangrene. The disorder may occur as a primary condition or secondary to infections, malignancies, autoimmune diseases, or systemic vasculitides (8–10).

**Figure 4:**
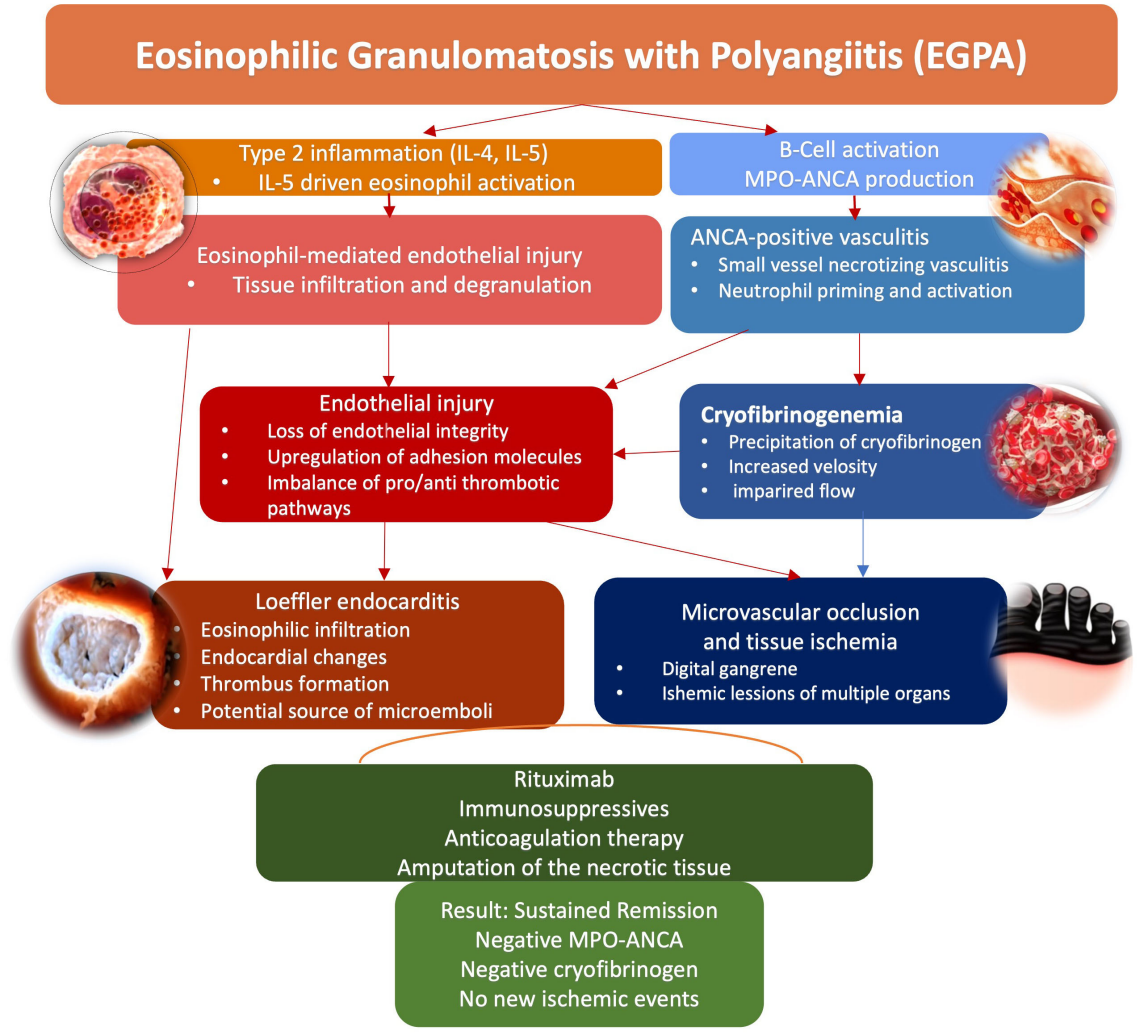
Proposed mechanisms potentially contributing to severe ischemic complications in EGPA.

In the present case, the clinical course suggests that secondary cryofibrinogenemia may have contributed to eosinophil-mediated endothelial injury, followed by autoantibody formation and subsequent precipitation of thrombogenic plasma proteins, resulting in inflammatory-thrombotic vasculopathy and rapid progression toward digital gangrene and multiorgan ischemia despite continuous anticoagulation and immunosuppressive therapy (**Figure 4**).

Cardiac manifestations represent one of the most serious complications of EGPA and are an important determinant of disease prognosis. Cardiac involvement occurs in approximately one-third of patients and is reported more frequently in ANCA-negative disease (13, 14). Eosinophilic infiltration of the myocardium leads to endomyocardial injury, which may result in thrombus formation, valvular abnormalities, and progressive fibrosis. Loeffler endocarditis represents the most severe form of eosinophilic cardiac involvement and may ultimately lead to restrictive cardiomyopathy (14, 15). Intracardiac thrombi can serve as a source of systemic embolization.

From an echocardiographic perspective, Loeffler endocarditis is characterized by diffuse endocardial thickening and layered, often filamentous thrombotic material, in contrast to more localized valvular lesions seen in nonbacterial thrombotic or Libman-Sacks endocarditis. Transesophageal echocardiography is particularly useful for detecting these changes.

In the present case, the visualization of “pearl-like” filamentous structures on transesophageal echocardiography, in the appropriate clinical context, strongly supported the diagnosis of eosinophilic endocardial involvement. These findings indicate a high-risk thrombo-inflammatory state with important therapeutic implications, supporting the need for intensified immunosuppressive and anticoagulant therapy. Advanced imaging modalities, particularly transesophageal echocardiography and cardiac magnetic resonance imaging, are essential for early detection of cardiac involvement and intracardiac thrombi (16).

## Neurological and systemic ischemic manifestations

Peripheral nervous system involvement is a common manifestation of EGPA and typically results from ischemic injury of the vasa nervorum caused by small-vessel vasculitis. The most characteristic presentation is mononeuritis multiplex, which may progress to sensorimotor polyneuropathy and can be confirmed by electrophysiological studies.

In ANCA-positive EGPA, neurological involvement is more frequently observed and reflects ANCA-mediated neutrophil activation leading to vascular inflammation and occlusion. The neurological manifestations observed in this patient are therefore consistent with the vasculitic phase of the disease.

The coexistence of ANCA-mediated vascular inflammation and cryofibrinogen-associated thrombosis likely contributed to the extensive ischemic burden affecting peripheral nerves and multiple organs.

## Therapeutic implications

Management of EGPA depends on the dominant pathogenic mechanism driving the disease. Eosinophilic manifestations are typically responsive to glucocorticoids and therapies targeting the IL-5 pathway, whereas ANCA-positive organ-threatening disease reflects B-cell–mediated autoimmunity, and usually requires remission-induction therapy with cyclophosphamide or B-cell depletion (17, 18). There is also a possibility of tissue injury shift from eosinophil-dominant inflammation toward autoantibody-driven vasculopathy. Recognition of this transition is clinically important, because IL-5-directed therapy alone may be insufficient once antibody-mediated vascular injury predominates.

In the present case, progression of ischemic complications despite corticosteroids, cyclophosphamide, plasmapheresis, and anticoagulation suggested ongoing antibody-mediated vascular injury. B-cell depletion with rituximab resulted in disappearance of MPO-ANCA and cryofibrinogenemia together with sustained clinical remission (18).

The parallel resolution of autoantibodies and thrombogenic plasma proteins supports a central role of adaptive immune activation in maintaining the severe disease phenotype. At the time therapeutic decisions were made, rituximab was not part of the usual treatment algorithm. In the later course, it was considered in severe ANCA positive forms of EGPA with predominant neurological, renal and gastrointestinal involvement with vasculitic complications. However, rituximab maintenance was less established than the current recommendations, and usually reserved for the therapeutically refractory course with frequent exacerbations, which was not the case for our patient.

Conventional immunosuppressives, such as methotrexate or azathioprine are typically selected as conventional remission maintenance therapy. However, due to noted hepatotoxicity, borderline leucopenia and hypogammaglobulinemia, the immunosuppressives of choice became the antimalarial medication, that sustained clinical remission so far. Eosinophil count is always closely monitored, keeping in mind the potential use of IL-5–directed therapy during disease relapse characterized by eosinophilic activity. IL-5–targeted therapy may be introduced and can serve as an effective long-term maintenance strategy (17).

## Limitations

Several limitations should be acknowledged. First, only semiquantitative testing for cryofibrinogenemia was available at the moment, and histopathological confirmation was not obtained. Second, the patient received only two doses of cyclophosphamide before escalation to rituximab, due to noted cardiovascular involvement and disease progression, so it cannot be definitively interpreted as cyclophosphamide refractoriness. Third, although extensive thrombophilia screening and antiphospholipid antibody testing were negative, alternative thrombotic mechanisms cannot be completely excluded, including the seronegative antiphospholidsyndrome. Therefore, direct pathogenic role cannot be established, but only hypothesized given that severe form of EGPA can lead to observed ischemicmanifestations.

## Conclusion

This case describes rare and severe presentation of EGPA characterized by the coexistence of eosinophilic cardiac involvement (Loeffler endocarditis), MPO-ANCA-mediated vasculitis and secondary cryofibrinogenemia leading to digital gangrene.

The clinical course suggests that endothelial injury induced by eosinophils and ANCA-activated neutrophils may promote cryofibrinogen precipitation, resulting in a combined inflammatory-thrombotic vasculopathy.

Disease progression despite conventional immunosuppressive therapy indicated a shift from eosinophilic inflammation toward antibody-mediated vascular injury, while sustained remission following B-cell depletion supports the central role of adaptive immune activation in this severe phenotype.

Recognition of overlapping inflammatory and thrombotic mechanisms is therefore essential for timely diagnosis and mechanism-based treatment selection in patients with severe EGPA.

## Data Availability

The original contributions presented in the study are included in the article/supplementary material. Further inquiries can be directed to the corresponding author.
